# Supplementary material for: Fitness effects of CRISPR endonucleases in Drosophila melanogaster populations
Source: eLife. 2022 Sep 22;11:e71809. doi: 10.7554/eLife.71809 (PMC9545523; doi:10.7554/eLife.71809)
Supplement: Supplementary file 1. — All cut parameters were set to 1. [file elife-71809-supp1.docx]

Supplementary File 1. Model comparison of Cas9_no-gRNAs, no-Cas9_no-gRNAs, Cas9HF1_gRNAs. All cut parameters were set to 1

| construct | model | selection | $\hat{N}_{e}$ | direct  fitness estimate | off-target  fitness  estimate | $ln\hat{L}$ | *P* | AICc |
| --- | --- | --- | --- | --- | --- | --- | --- | --- |
| Cas9_no-gRNAs | full | viability | 252  [157 -379] | 1  [0.97 – 1.05] | 0.76  [0.57 – 1.29] | 89.3 | 3 | -171 |
| Cas9_no-gRNAs | construct | viability | 243  [152 - 366] | 1  [0.96 – 1.04] | 1* | 88.6 | 2 | -173 |
| Cas9_no-gRNAs | off-target | viability | 252  [157 - 379] | 1* | 0.76  [0.57 – 1.29] | 89.3 | 2 | -174 |
| Cas9_no-gRNAs | neutral | none | 243  [152 – 366] | 1* | 1* | 88.6 | 1 | -175 |
| no-Cas9_no-gRNAs | full | viability | 162  [101 - 244] | 1  [0.97 -1.10] | 1.06  [0. 74 – 2.06] | 81.5 | 3 | -155 |
| no-Cas9_no-gRNAs | construct | viability | 162  [101 - 244] | 1  [0.97 -1.10] | 1* | 81.5 | 2 | -158 |
| no-Cas9_no-gRNAs | off-target | viability | 162  [101 - 244] | 1* | 1.06  [0. 74 –2.06] | 81.5 | 2 | -158 |
| no-Cas9_no-gRNAs | neutral | none | 162  [101 - 244] | 1* | 1* | 81.5 | 1 | -161 |
| Cas9HF1_gRNAs | full | viability | 444  [240 – 682] | 0.99  [0.96 – 1.02] | 1.35  [1.04 – 1.97] | 90.2 | 3 | -173 |
| Cas9HF1_gRNAs | construct | viability | 396  [240 - 608] | 1  [0.97 – 1.04] | 1* | 88.1 | 2 | -171 |
| Cas9HF1_gRNAs | off-target | viability | 440  [267 – 675] | 1* | 1.30  [1.00 – 1.88] | 90.0 | 2 | -175 |
| Cas9HF1_gRNAs | neutral | none | 396  [240 - 608] | 1* | 1* | 88.1 | 1 | -174 |

Each row shows the parameter estimates ($\hat{N}_{e}$= effective population size), maximum log Likelihood (ln$\hat{L})$, number of free parameters in the maximum likelihood framework (*P*), and corrected Akaike Information Criterion value ($AICc=2p-2ln\hat{L}+(2p^{2}+2p)/(n-p-1$) where *n* = number of generation transitions; *n* = 20 for Cas9_no-gRNAs, no-Cas9_no-gRNAs, and *n* = 18 for Cas9HF1_gRNAs) for a specific construct, model and selection type. 1* entries indicate that a parameter was fixed at 1 (= no fitness effect is estimated). Values in squared brackets in the parameter estimate columns represent the 95 % confidence intervals estimated from a likelihood ratio test with one degree of freedom.
