# Supplementary material for: Fitness effects of CRISPR endonucleases in Drosophila melanogaster populations
Source: eLife. 2022 Sep 22;11:e71809. doi: 10.7554/eLife.71809 (PMC9545523; doi:10.7554/eLife.71809)
Supplement: Supplementary file 2. — Lowercase letters indicate a mismatch between the potential off-target sequence and the gRNA sequence. [file elife-71809-supp2.docx]

Supplementary File 2. Predicted off-target sequences for gRNAs 1 to 4. Lowercase letters indicate a mismatch between the potential off-target sequence and the gRNA sequence.

| gRNA | target sequence  off-target sequence | off-target position | off-target annotation |
| --- | --- | --- | --- |
| 1 | AGTGGAAAACGGAGACCACC GGG  caatcAgAACGGAGACCACC AGG  AGTttAcggCGGAGACCACC AGG | 3L: 11473623 – 11473645  3R: 30428889 – 30428911 | *vhA16-3* gene, coding sequence  *CG5533* gene, coding sequence |
| 2 | AGGGACAGAACTTGCGGGGA GGG  AGGtACgcAACTTGCGGGaA AGG  AcaaACtGAAaTTGCGGGGA AGG | 2R: 11951576 –11951598  3R: 31879082 – 31879104 | *exp* gene, deep within intron  *heph* gene, deep within intron |
| 3 | TAAACCAATTTATACCGAGT CGG  cgAAggAAaTTATACCGAGT CGG  TAAACtAATTaAaACCGAGT GGG | 2R: 8862838 – 8862860  3R: 24565312 – 24565334 | *RyR* gene, 5’UTR  *REPTOR* gene, deep within intron |
| 4 | GGCAATATATAGGAATGCAC TGG  no predicted off-targets |  |  |
