# Supplementary material for: Fitness effects of CRISPR endonucleases in Drosophila melanogaster populations
Source: eLife. 2022 Sep 22;11:e71809. doi: 10.7554/eLife.71809 (PMC9545523; doi:10.7554/eLife.71809)
Supplement: Supplementary file 3. — For each construct, DNA fragments, plasmids, primers, and restriction enzymes used for cloning are listed. [file elife-71809-supp3.docx]

**Plasmid construction overview**

Construct with just DsRed (“no-Cas9_no-gRNAs”):

| **FACacR** | *Template* | *Oligo/Enzyme 1* | *Oligo/Enzyme 2* |
| --- | --- | --- | --- |
| *PCR Product* | pDsRed | FACacR_F | FACacR_R |
| *Plasmid Digest* | ATSacG | NcoI | HincII |

Intermediate for the gRNAs:

| **TTTacU4** | *Template* | *Oligo/Enzyme 1* | *Oligo/Enzyme 2* |
| --- | --- | --- | --- |
| *PCR Product* | TTTgRNAtRNAi | Acg4_41_F | Acg4_41_R |
| *PCR Product* | TTTgRNAt | Acg4_12_F | Acg4_12_R |
| *PCR Product* | TTTgRNAt | Acg4_23_F | Acg4_23_R |
| *PCR Product* | TTTgRNAt | Acg4_34_F | Acg4_34_R |

Construct with Cas9 and no gRNAs (“Cas9_no-gRNAs”):

| **FACacN** | *Template* | *Oligo/Enzyme 1* | *Oligo/Enzyme 2* |
| --- | --- | --- | --- |
| *PCR Product* | none | acN_F | acN_R |
| *Plasmid Digest* | BHDgN1c | StuI | XbaI |

Construct with Cas9 and four gRNAs (“Cas9_gRNAs”):

| **FACacN4** | *Template* | *Oligo/Enzyme 1* | *Oligo/Enzyme 2* |
| --- | --- | --- | --- |
| *PCR Product* | none | U6_3_gRNA1_v4_F | gRNA_f_R |
| *Plasmid Digest* | BHDgN1c | StuI | XbaI |

Intermediate for Cas9HF1:

| **Nos-Cas9HF1** | *Template* | *Oligo/Enzyme 1* | *Oligo/Enzyme 2* |
| --- | --- | --- | --- |
| *PCR Product* | VP12 | Cas9HF1_F | Cas9HF1_R |
| *Plasmid Digest* | nos-Cas9-nos | Bsu36I | FspI |

Construct with Cas9HF1 and four gRNAs (“Cas9HF1_gRNAs”):

| **FACacNf4** | *Template* | *Oligo/Enzyme 1* | *Oligo/Enzyme 2* |
| --- | --- | --- | --- |
| *PCR Product* | Nos-Cas9HF1 | HF1_F | HF1_R |
| *Plasmid Digest* | FACacN4 | Bsu36I | AscI |

Homing drive with Cas9HF1:

| **BHDgNf1v2** | *Template* | *Oligo/Enzyme 1* | *Oligo/Enzyme 2* |
| --- | --- | --- | --- |
| *PCR Product* | Nos-Cas9HF1 | HF1_F | HF1_R |
| *Plasmid Digest* | BHDgN1cv3 | Bsu36I | AscI |

**Construction oligonucleotides**

Acg4_12_F: GGCAATATATAGGAATGCACGTTTTAGAGCTAGAAATAGCAAGTTAAA

Acg4_12_R: AACACTCGGTATAAATTGGTTTATGCACCAGCCGGGAATCG

Acg4_23_F: GCATAAACCAATTTATACCGAGTGTTTTAGAGCTAGAAATAGCAAGTTAAA

Acg4_23_R: AACTCCCCGCAAGTTCTGTCCCTTGCACCAGCCGGGAATCG

Acg4_34_F: GCAAGGGACAGAACTTGCGGGGAGTTTTAGAGCTAGAAATAGCAAGTTAAA

Acg4_34_R: GGTGGTCTCCGTTTTCCACTTGCACCAGCCGGGAATCG

Acg4_41_F: GTGCAAGTGGAAAACGGAGACCACCGTTTTAGAGCTAGAAATAGCAAGTTAAA

Acg4_41_R: AAAACGTGCATTCCTATATATTGCCTGCATCGGCCGGGAATCG

acN_F: CAAACTCATCAATGTATCTTAACCGGTAGGAGCAAGCTGCCCGTGCCCTGGCCCACCCTC

acN_R: GAGGGTGGGCCAGGGCACGGGCAGCTTGCTCCTACCGGTTAAGATACATTGATGAGTTTG

Cas9HF1_F: CACCTGGGCGAACTGCACGCTATCCTCAGGAGGCAGGAGGATTTTTATCCGT

Cas9HF1_R: ACCACTGCATTCAGGTAGGCATCATGCGCATGGTGGTAGTTATTTATCTCCCTAACTTT

FACacR_F: CTAAACAATCGGCTCGAAGC

FACacR_R: GTAACCATTATAAGCTGCAATAAACAA

gRNA_f_R: GAGGGTGGGCCAGGGCACGGGCAGCTTGCTCTAGAATGCATACGCATTAAGCGAACA

HF1_F: GGTGGTGTCGAAGTACTTGAAG

HF1_R: AGATTCACCTGGGCGAACTG

U6_3_gRNA1_v4_F: GTCCAAACTCATCAATGTATCTTAACCGGTAGGCCTTTTTTTGCTCACCTGTGATTGCTC

**Sequencing oligonucleotides**

AutoDLeft_S2_F: CTTACGCTGAAGCCATTTCAA

AutoDRight_S2_R: ATCTGGTTCTCACTTCCATTTAAAT

Cas9_HF1_S1_R: GGACTTTCTTGTCATCCATGCG

Cas9HF1_S_R: CTACCCCGGAGATCTCGACAG

Cas9mid_S_F: CGACCAGTACGCAGACCTTTT

DsRed_S_F: CTGAAGGGCGAGATCCACAAG

EGFP_S_R: AGTTGTACTCCAGCTTGTGCC

pCFD5_S_R: ACGTCAACGGAAAACCATTGTCTA

AutoC_S2_F: TCAAGCCATGTGACCCAGATT

EGFPaLeft_S_R: GCTTGTTTATTTGCTTAGCTTTCGC
