# Supplementary figures and images for: Fitness effects of CRISPR endonucleases in Drosophila melanogaster populations
Source: eLife. 2022 Sep 22;11:e71809. doi: 10.7554/eLife.71809 (PMC9545523; doi:10.7554/eLife.71809)

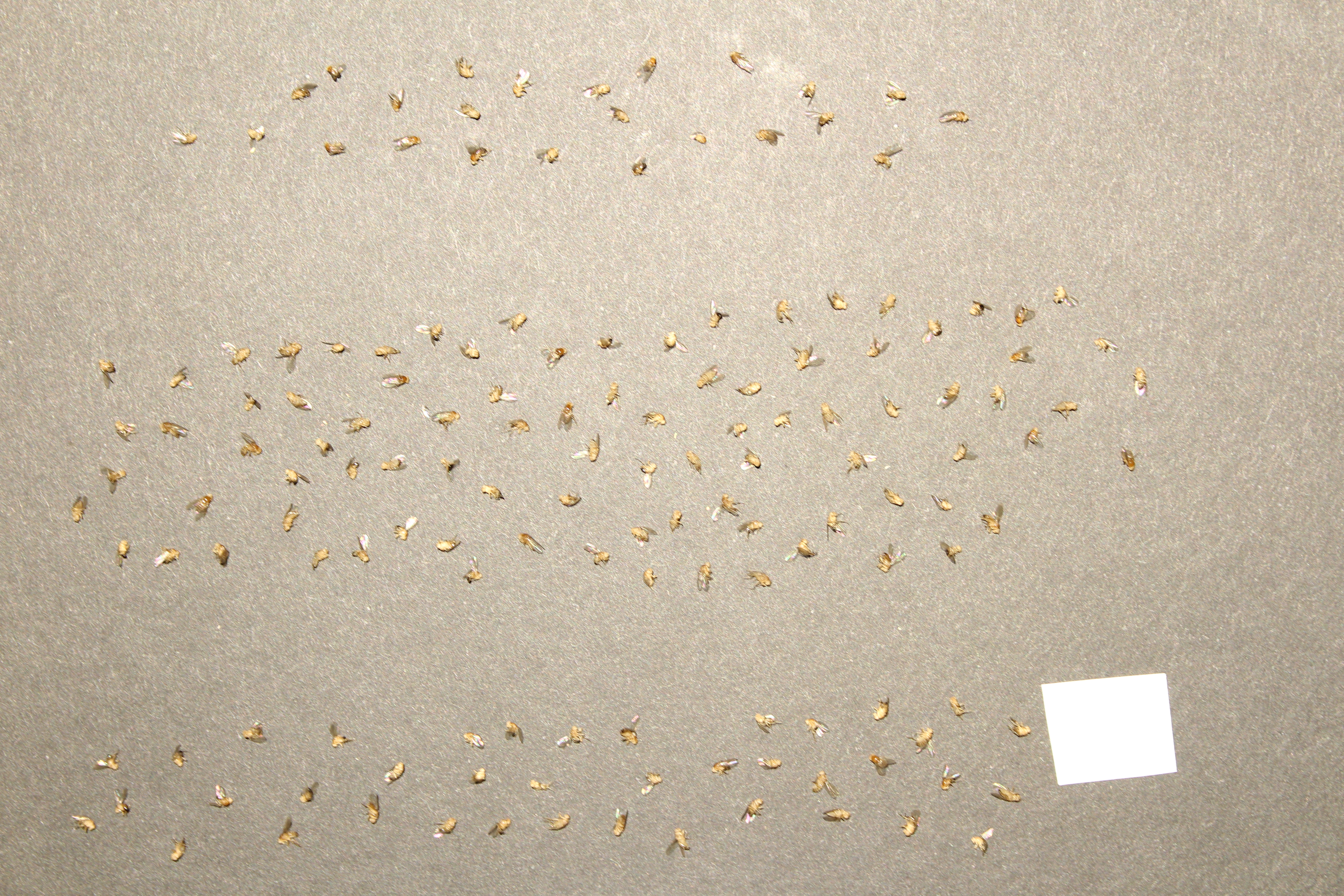

Supplement: Supplementary file 6. [file elife-71809-supp6.zip › example-pictures/IMG_0001.JPG]

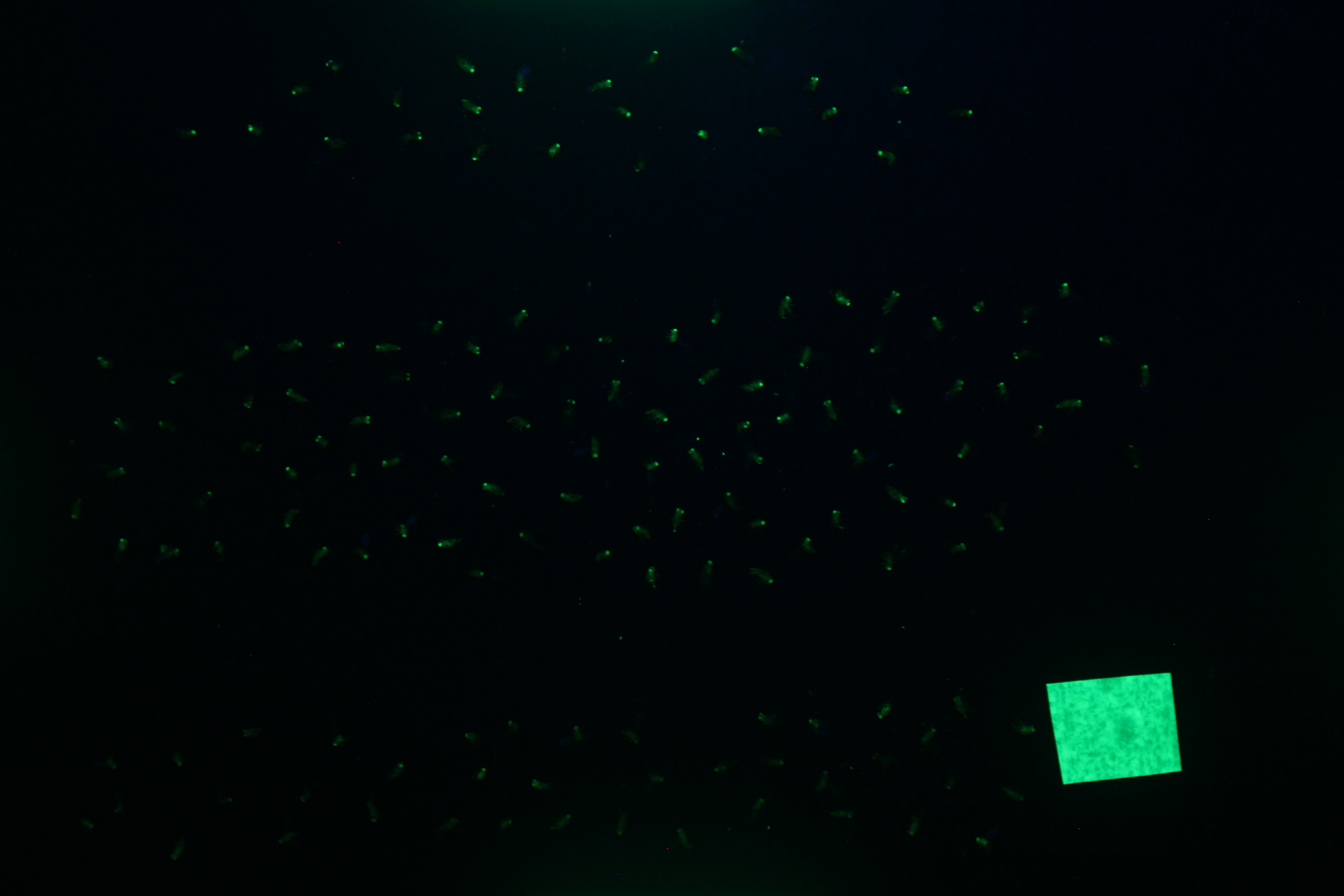

Supplement: Supplementary file 6. [file elife-71809-supp6.zip › example-pictures/IMG_0003.JPG]

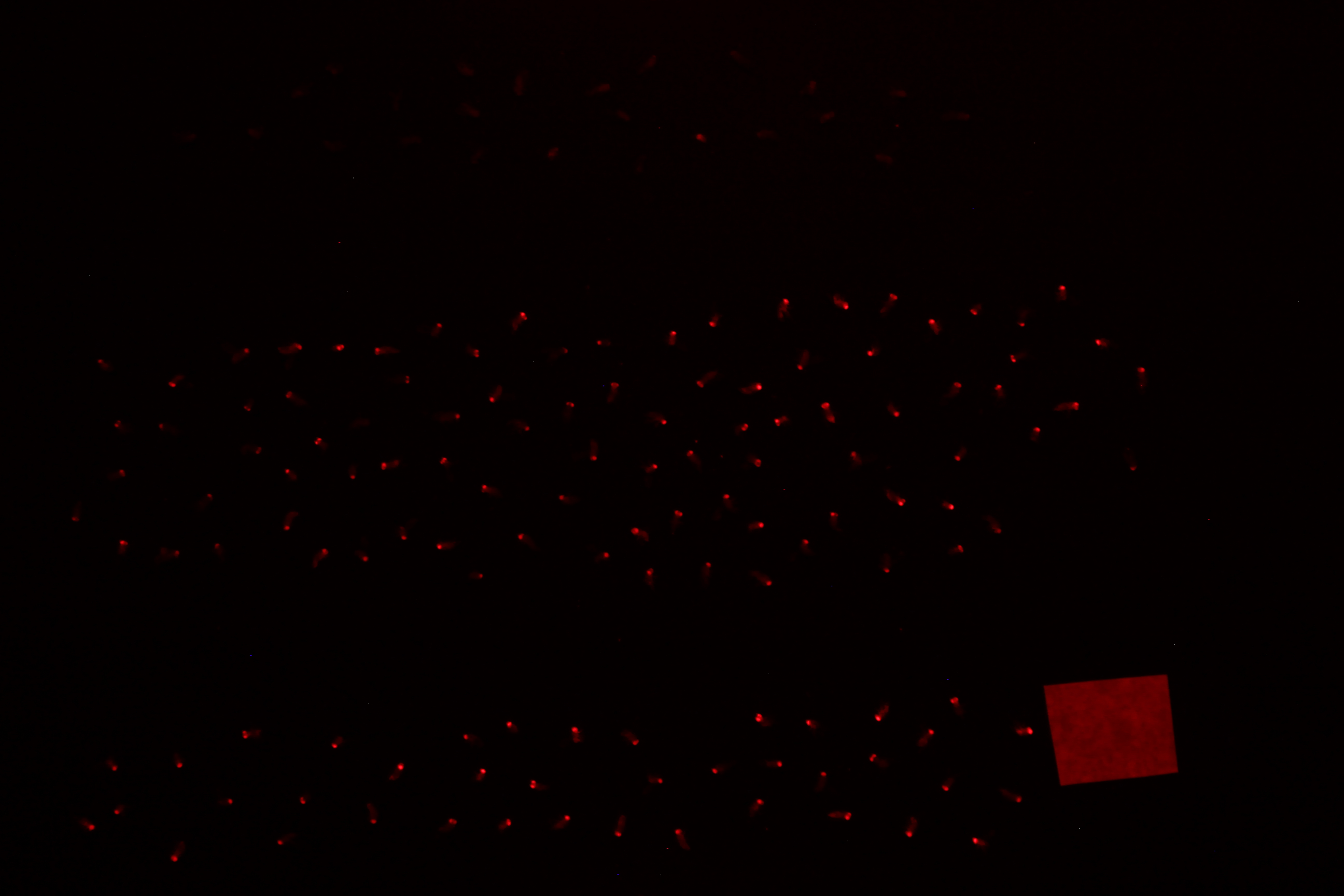

Supplement: Supplementary file 6. [file elife-71809-supp6.zip › example-pictures/IMG_0002.JPG]
