# Supplementary material for: Fitness effects of CRISPR endonucleases in Drosophila melanogaster populations
Source: eLife. 2022 Sep 22;11:e71809. doi: 10.7554/eLife.71809 (PMC9545523; doi:10.7554/eLife.71809)
Supplement: Transparent reporting form [file elife-71809-transrepform1.docx]

***eLife’s* transparent reporting form**

We encourage authors to provide detailed information *within their submission* to facilitate the interpretation and replication of experiments. Authors can upload supporting documentation to indicate the use of appropriate reporting guidelines for health-related research (see [EQUATOR Network](http://www.equator-network.org/%20)), life science research (see the [BioSharing Information Resource](https://biosharing.org/)), or the [ARRIVE guidelines](http://www.plosbiology.org/article/info:doi/10.1371/journal.pbio.1000412) for reporting work involving animal research. Where applicable, authors should refer to any relevant reporting standards documents in this form.

If you have any questions, please consult our Journal Policies and/or contact us: [editorial@elifesciences.org](mailto:editorial@elifesciences.org).

**Sample-size estimation**

- You should state whether an appropriate sample size was computed when the study was being designed
- You should state the statistical method of sample size computation and any required assumptions
- If no explicit power analysis was used, you should describe how you decided what sample (replicate) size (number) to use

Please outline where this information can be found within the submission (e.g., sections or figure legends), or explain why this information doesn’t apply to your submission:

Sample sizes in individual cages were set to allow a maximum number of flies per cage while still keeping an appropriate amount of working space. This fixed the number of food bottles at eight per cage, which in turn determined the population/sample size (Section “Experimental fly populations” Methods section, line 762– 788).

For experiments with individual crosses (phenotypic assays, Cas9HF1 homing drive), sample size was based on the total number of flies phenotyped, usually calibrated to give binomial errors of a few percent at most, which we considered suitable for drawing conclusions.

**Replicates**

- You should report how often each experiment was performed
- You should include a definition of biological versus technical replication
- The data obtained should be provided and sufficient information should be provided to indicate the number of independent biological and/or technical replicates
- If you encountered any outliers, you should describe how these were handled
- Criteria for exclusion/inclusion of data should be clearly stated
- High-throughput sequence data should be uploaded before submission, with a private link for reviewers provided (these are available from both GEO and ArrayExpress)

Please outline where this information can be found within the submission (e.g., sections or figure legends), or explain why this information doesn’t apply to your submission:

Number of replicates for population cage experiments: Section “Experimental fly populations” in Methods section (line 762 – 788)

Outlier/exclusion of data for population cage experiments: Section “Experimental fly populations” in Method section (line 762 – 788)

Number of replicates for phenotypic assays: Section “Phenotypic assays” in Methods section (line 842 – 884)

Number of replicates for Cas9HF1 homing gene drive – phenotype data analysis: Supplementary File 5 (Data Set S1-S2)

Data obtained are provided as supplementary files (Section “Availability of data and materials”, line 1000 – 1010).

Outlier/exclusion of data for phenotypic assays: Section “Phenotypic fitness assays” in Result section (Mate choice assay; Line 353 – 360)

**Statistical reporting**

- Statistical analysis methods should be described and justified
- Raw data should be presented in figures whenever informative to do so (typically when N per group is less than 10)
- For each experiment, you should identify the statistical tests used, exact values of N, definitions of center, methods of multiple test correction, and dispersion and precision measures (e.g., mean, median, SD, SEM, confidence intervals; and, for the major substantive results, a measure of effect size (e.g., Pearson's r, Cohen's d)
- Report exact p-values wherever possible alongside the summary statistics and 95% confidence intervals. These should be reported for all key questions and not only when the p-value is less than 0.05.

Please outline where this information can be found within the submission (e.g., sections or figure legends), or explain why this information doesn’t apply to your submission:

Description of Maximum likelihood framework (fitness cost quantification): Section “Maximum likelihood analysis” in Results (line 192 – 202, 235 – 254) and “Maximum Likelihood framework for fitness cost estimation” in Methods section (line 927 – 978)

Description of statistical methods (phenotypic assays): Section “Phenotypic assays” in Methods (line 842 – 884) & Results (line 348 –415, Figure 4)

Description of statistical methods & statistical tests used (Cas9HF1 homing gene drive): Methods section “phenotype data analysis, Cas9HF1 homing gene drive”, line 886 – 902) & Supplementary File 5 & Section “Cas9HF1 homing drive” in Results (line 436– 481)

Raw data is presented in: Figure 2,Figure 2- figure supplement1, Figure 3, Figure 3-figure supplement2, Figure 5; Figure 4 has N>10 per group; other Figures contain either only simulated data or are conceptional.

Statistical tests used (phenotypic assays): caption Figure 4 + Section “Phenotypic assays” in Methods section (line 842 – 884)

Exact P-values (phenotypic assays): Figure 4 contains all P-values for each statistical test conducted; Multiple testing correction is described in the caption of Figure 4

Exact P-values (Cas9HF1 homing gene drive): Section “Cas9HF1 homing drive” in Results (line 436 – 481)

(For large datasets, or papers with a very large number of statistical tests, you may upload a single table file with tests, Ns, etc., with reference to sections in the manuscript.)

**Group allocation**

- Indicate how samples were allocated into experimental groups (in the case of clinical studies, please specify allocation to treatment method); if randomization was used, please also state if restricted randomization was applied
- Indicate if masking was used during group allocation, data collection and/or data analysis

Please outline where this information can be found within the submission (e.g., sections or figure legends), or explain why this information doesn’t apply to your submission:

This does not apply since each experiment required specific fly genotypes, which required specific allocation by researchers.

**Additional data files (“source data”)**

- We encourage you to upload relevant additional data files, such as numerical data that are represented as a graph in a figure, or as a summary table
- Where provided, these should be in the most useful format, and they can be uploaded as “Source data” files linked to a main figure or table
- Include model definition files including the full list of parameters used
- Include code used for data analysis (e.g., R, MatLab)
- Avoid stating that data files are “available upon request”

Please indicate the figures or tables for which source data files have been provided:

Raw data are provided as Supplementary files (Section “Availability of data and materials”, line 1000 – 1010).

Code used for data analysis is available on GitHub (Section “Availability of data and materials”, line 1000 – 1010& Section “Phenotype data analysis, Cas9HF1 homing gene drive” in Material and Methods, line 886 – 902).
